# 1例3次挑战培美曲塞方案治疗晚期非小细胞肺癌有效的个案报道

**DOI:** 10.3779/j.issn.1009-3419.2019.06.11

**Published:** 2019-06-20

**Authors:** 林林 程, 峨嵋 高, 富新 朱, 玉艳 王, 佳 仲, 彤同 安

**Affiliations:** 1 252200 聊城，东阿县人民医院 Dong'e County Hospital, Liaocheng 252200, China; 2 100142 北京，北京大学肿瘤医院 Peking University Cancer Hospital, Beijing 100142, China

**Keywords:** 肺肿瘤, 培美曲塞, 再次挑战, 三次挑战, Lung neoplasms, Pemetrexed, Re-challenge, The third challenge

## Abstract

非小细胞肺癌（non-small cell lung cancer, NSCLC）约占肺癌的85%，5年总生存率小于15%-19%，且80%以上的肺癌患者明确诊断时属于中晚期（Ⅲb期-Ⅳ期），以化疗为主的综合治疗是目前无驱动基因突变的晚期NSCLC的主要治疗方式。以培美曲塞为基础的含铂两药方案及培美曲塞单药方案，分别是一线主要指南推荐方案及二线可选择方案，而三线及后线治疗无循证医学依据，根据患者既往用药情况选择后线治疗方案是临床常用的重要方法。培美曲塞是高效低毒的多靶点化疗药物，晚期NSCLC再挑战应用培美曲塞方案是一种合理的选择。本文报道1例三次挑战使用培美曲塞基础方案治疗晚期NSCLC有效的个案病例并做相关文献复习。

## Abstract (en)

Non-small cell lung cancer (NSCLC) accounts for about 85% of lung cancer, with a 5-year survival rate of less than 15%-19%, and more than 80% of the patients with lung cancer have progressed to advanced stage (Stage Ⅲb-Ⅳ) when they are clearly diagnosed. The comprehensive treatment mainly based on chemotherapy as the primary form is now considered as the major therapy method for advanced NSCLC without actionable driver gene mutations. Pemetrexed plus platinum doublet as well as single-agent pemetrexed are respectively the first-line major regimens recommended by guidelines and the second-line optional regimens. Yet the third-line treatment or beyond in advanced NSCLC is not evidence-based but conducted based on patients' previous medications, which is one of the most commonly used clinical methods. As pemetrexed is a multi-target chemotherapy drug with high efficiency but low toxicity, pemetrexed re-challenge strategy in advanced NSCLC is also a reasonable choice. We report one effective individual case that adopted pemetrexed re-challenge strategy in advanced NSCLC for three times, and at the same time conduct the relevant literature review.

## 1 临床资料

女性患者，61岁，患者于2015年10月无诱因出现间断性咳嗽，咳白色粘痰，2015年11月6日，胸部计算机断层扫描（computed tomography, CT）显示：右肺上叶前段见软组织肿块，呈分叶状，约44 mm×35 mm，与肺门血管关系密切，伴有阻塞性肺炎，纵隔2组-4组有多发肿大淋巴结，较大者约20 mm×15 mm，右肺门肿大淋巴结，约18 mm×16 mm，右侧胸膜有弥漫结节样增厚，并伴有右侧胸腔积液。2015年10月27日，纤维支气管镜显示：右肺上叶尖段狭窄，刷片可见腺癌细胞；右侧胸水涂片：可见腺癌，胸水*EGFR*、*KRAS*呈野生型，头磁共振成像（magnetic resonance imaging, MRI）未见异常。超声显示：右颈根有0.7 cm×0.5 cm淋巴结，左肾囊肿，其余未见异常。全身骨扫描显示：左侧肩胛骨骨盐代谢旺盛灶，考虑为骨转移瘤，T7骨盐代谢增高，结合胸部CT骨窗除外转移，L5双侧、右踝关节骨盐代谢旺盛灶，考虑为良性病变。初步诊断为右肺腺癌cT2N2M1 Ⅳ期，纵隔淋巴结转移，右侧恶性胸腔积液，胸水包埋EGFR野生型，KRAS野生型，ALK-ventana（-）骨转移待除外。

患者自2015年11月4日行一线培美曲塞+顺铂方案化疗4个周期，具体方案为：培美曲塞500 mg/m^2^，d1，顺铂75 mg/m^2^，d1（顺铂分两日输注）；*Q21d*，末次化疗时间：2016年1月11日。第1周期化疗期间引流右侧胸水1, 600 mL后，给予胸腔灌注贝伐珠单抗0.1 g，化疗耐受性可。2周期评效缩小，疾病稳定（stable disease, SD）（缩小11.3%），4周期评效缩小SD（与基线比较，缩小13.4%）。2016年2月3日-2016年6月12日，行培美曲塞单药维持化疗7个周期，维持治疗4个周期后评效部分缓解（partial response, PR）（与基线相比，缩小33%），维持治疗6个周期评效增大SD，维持治疗7个周期后评效PD（右肺上叶前段肿块较前增大，29 mm×21 mm至42 mm×33 mm），无进展生存期（progression free survival, PFS）9.6个月。

患者自2016年7月12日行二线多西他赛（泰索帝）方案治疗6个周期。具体方案是：多西他赛75 mg/m^2^，d1；*Q21d*；末次化疗时间：2016年10月31日。化疗过程后出现脱发2度、中性粒细胞减少4度（0.7×10^9^/L），自行恢复正常。2周期评效为缩小SD(胸腔积液消失，胸部病灶稍缩小)，4周期评效为缩小SD（右肺病灶稍缩小），6周期评效为SD。患者于2017年2月7日进行基线检查，显示右肺上叶前段肿块较前增大（35 mm×18 mm至48 mm×37 mm），头颅增强MR未见转移征象，综合评效PD，PFS 6.8个月。

考虑患者既往培美曲塞+顺铂方案获益时间较久，三线再挑战含培美曲塞双药方案，自2017年2月15日开始行三线线培美曲塞+顺铂方案化疗4个周期。具体方案：培美曲塞500 mg/m^2^，d1，顺铂75 mg/m^2^，d1（顺铂分两日输注）；*Q21d*。末次化疗时间：2017年4月22日。化疗耐受性可，2个周期评效为缩小SD，4个周期评效为SD（变化不大），之后定期复查。2017年7月，患者稍感轻度憋气，余无不适，并于2017年7月17日复查胸部CT，发现右肺上叶癌、右侧胸膜转移较前进展（右肺上叶肿物33 mm×26 mm至45 mm×36 mm），脑磁共振成像MRI、腹盆计算机断层扫描CT、浅表淋巴结彩超无转移征象。考虑肿瘤进展，PFS 5.0个月。

四线入组免疫治疗临床试验：分别于2017年8月10日、2017年8月31日、2017年9月21日，给予四线IBI308（信达PD-1单抗）免疫治疗3周期，评效PD（增大60%，右肺上叶肿物49 mm×38 mm至78 mm×67 mm，远端肺不张加重、上叶完全实变，肿瘤标志物明显升高），退出免疫治疗，PFS 3个月。

患者四线治疗进展，五线第三次挑战培美曲塞，考虑既往未用贝伐珠单抗，五线加用贝伐珠单抗，自2017年10月14日开始给予五线培美曲塞+顺铂+贝伐珠单抗方案化疗6个周期，具体方案是：培美曲塞500 mg/m^2^，d1，顺铂75 mg/m^2^，d1（顺铂分两日输注），贝伐珠单抗7.5 mg/kg，d1；*Q21d*。末次化疗时间：2018年2月1日。总体化疗耐受较好，无明显骨髓抑制及消化道反应。2个周期缩小SD（缩小约27.4%，接近PR），4个周期综合评效为PR（较基线缩小39.6%，较2个周期治疗后缩小12.8%）。6周期治疗后维持PR，继行培美曲塞+贝伐珠单抗方案维持治疗2个周期。2018年4月26日综合评效为PD，PFS：6.4个月。

患者既往史：既往2型糖尿病病史10年，规律口服二甲双胍、罗格列酮降糖治疗，血糖控制可；既往高血压病史3年，最高140 mmHg/90 mmHg，长期口服硝苯地平片降压治疗，血压控制可；应用贝伐珠单抗期间血压监测正常。个人史：否认嗜酒史、吸烟史。家族史：否认家族性肿瘤病史。

## 2 相关文献学习及讨论

非小细胞肺癌是常见的恶性肿瘤，80%以上的肺癌患者明确诊断时属于中晚期（Ⅲb期-Ⅳ期），对于无驱动基因突变、无靶向治疗选择的转移性非小细胞肺癌的患者，或者靶向治疗后进展、免疫治疗效果不佳的患者，化疗是主要的治疗方式^[1]^。培美曲塞是近年研发并兴起的新型多靶点抗代谢化疗药物，是一种结构上含有核心为吡咯嘧啶基团的抗叶酸制剂，通过破坏细胞内叶酸依赖性的正常代谢过程，抑制细胞复制，从而抑制肿瘤的生长。体外研究表明，与传统化疗药作用于单一的酶不同，培美曲塞能够抑制胸苷酸合成酶、二氢叶酸还原酶和甘氨酰胺核苷酸甲酰转移酶一系列多种酶活性，可增强药物疗效，且不易产生耐药性，通过抑制叶酸的合成，从而抑制胸腺嘧啶核苷酸和嘌吟核苷酸的生物再合成过程，达到较好的抗肿瘤效果^[2]^。培美曲塞作为高效低毒的治疗药物，在临床应用中，成为一线治疗进展后，二线治疗再次挑战应用的重要选择方案。

Kaira等^[3]^在2010年对106例吉非替尼失败后使用厄洛替尼治疗的患者进行荟萃分析，DCR能达到28.8%，且发现口服厄洛替尼有效超过6个月的进展患者，使用厄洛替尼临床获益的可能性更大，因此，首次提出了再次挑战的概念。一些国外学者在恶性胸膜间皮瘤的回顾性分析中，发现再挑战应用培美曲塞的疗效可能与初始治疗的疗效相似，或者说，初始维持有效时长较长者，再次应用更易获得较好疗效。如2011年Ceresoli^[4]^、2012年Zucali PA^[5]^、2013年Shahid等^[6]^的相关报道。

**1:**
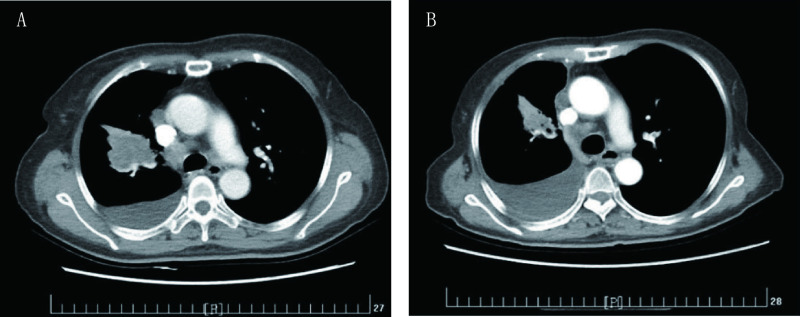
第一次培美曲塞应用。A：第一次应用前基线检查（2015年11月5日）；B：第一次应用最佳疗效（2016年3月4日） The first pemetrexed challenge. A: The baseline CT image before the first pemetrexed challenge (Nov. 5, 2015); B: The best response CT image after the first pemetrexed challenge (Mar. 4, 2016)

**2:**
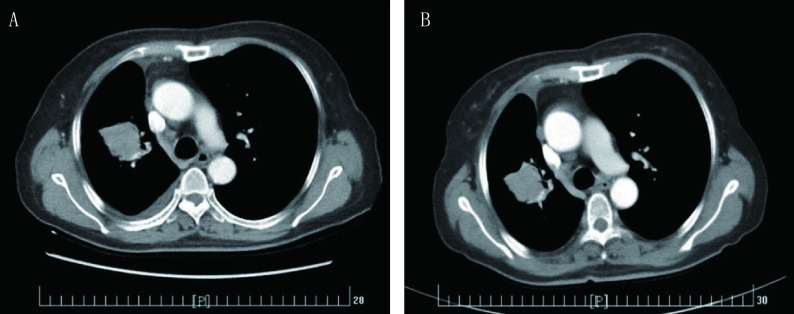
第二次培美曲塞应用。A：第二次应用前基线检查（2017年2月7日）；B：第二次应用最佳疗效（2017年3月28日） The second pemetrexed challenge. A: The baseline CT image before the second pemetrexed challenge (Feb. 7, 2017); B: The best response CT image after the second pemetrexed challenge (Mar. 28, 2017)

**3:**
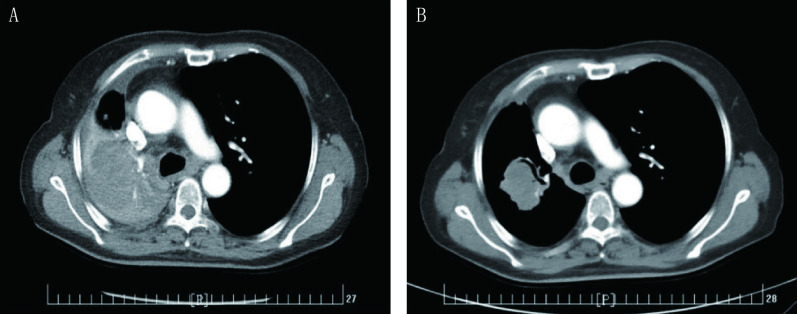
第三次培美曲塞应用。A：第三次应用前基线检查（2017年10月9日）；B：第三次应用最佳疗效（2018年1月2日） The third pemetrexed challenge. A: The baseline CT image before the third pemetrexed challenge (Oct. 9, 2017); B: The best response CT image after the third pemetrexed challenge (Jan. 2, 2018)

关于一线应用含培美曲塞方案治疗后进展明显，二线再次应用含培美曲塞方案的单中心回顾性研究分析已有多篇文献报道，而三次再挑战、尤其免疫治疗失败后，培美曲塞联用抗血管生成药物再挑战的临床病例较为罕见。本例即为三次挑战且多线后挑战培美曲塞方案成功的案例。

近年来，国内学者对晚期非小细胞肺癌患者二线或多线治疗失败后，再次挑战培美曲塞方案的尝试报道较多：2014年卓明磊等^[7]^回顾了31例一线应用培美曲塞为基础方案进展的腺癌，二线再次应用培美曲塞为基础的方案，揭示再挑战疗效与一线应用疗效有一定的相关性，一线PFS（PFS1） > 10个月的患者比PFS < 10个月的患者显示更长的二线PFS（PFS2）及OS[mPFS2: (6.2±0.33) mo *vs* (3.1±0.26) mo, mOS: (16.47±3.2) mo *vs* (8.33±1.24) mo, *P*=0.005]；2016年4月我国学者姜金等^[8]^回顾性分析32例一线应用培美曲塞联合铂类方案治疗的晚期NSCLC患者，肿瘤进展后二线或后线再次应用含培美曲塞，疗效显示：肿瘤控制率为40.6%（13/32），一线PFS > 6个月的患者，再次挑战培美曲塞的PFS为2.6个月，一线PFS < 6个月的患者再挑战培美曲塞的PFS为1.1个月（*P*=0.029），不良反应均可耐受。

2017年我国上海学者Lan等^[9]^评估培美曲塞二钠再挑战治疗晚期肺腺癌中的地位，选择那些既往培美曲塞为基础的化疗最佳疗效达PR或SD的，且并非进展停疗、停用培美曲塞至少3个月的患者，再挑战应用培美曲塞。观察62例符合条件患者，发现初治一线应用培美曲塞方案的46例，二线应用者16例，初始疗效为：19例PR（30.6%），43例获得SD（69.4%）。再挑战治疗作为二线、三线或多线的比例分别为43.5%、37.1%、19.4%，再挑战治疗的中位PFS为3.9个月，mOS为8.2（95%CI: 5.0-11.3），并且发现，既往培美曲塞初始治疗后再挑战间隔时间不影响再挑战治疗的效果，而且再挑战治疗的耐受性较好，40%出现1级-2级毒性，6%出现3级-4级毒性，均可恢复。这些研究均验证了培美曲塞作为新型多靶点抗代谢药物的高效、低毒及可恢复的耐药性，进一步证实既往培美曲塞治疗有效或控制的晚期非小细胞肺癌患者再次应用培美曲塞的有效性，多方面说明，二线或多线后再挑战应用培美曲塞方案不失为一种合理的选择。

本例患者分别在第一、第三、第五线应用含培美曲塞方案。一线培美曲塞+顺铂方案最佳疗效为缩小的SD，PFS 9.6个月；三线培美曲塞+顺铂方案，最佳疗效SD，PFS 5.0个月；五线培美曲塞+顺铂+贝伐珠单抗方案，最佳疗效PR，PFS目前尚未达进展。五线治疗时经历了免疫治疗的失败，但加用了抗血管生成治疗，最佳疗效达PR，甚至优于一线、三线的近期疗效，原因值得进一步探索。

本案例展示的多线后应用含培美曲塞方案的疗效，不仅与临床应用培美曲塞的高效性、低耐药性的典型特征紧密相关外，还可能与培美曲塞联合贝伐珠单抗抗血管生成治疗的协同作用相关。正常状态下肿瘤细胞会释放大量VEGF，当与相关受体结合后，刺激肿瘤新生血管形成^[10]^。当贝伐珠单抗与VEGF相结合，会抑制VEGF的活性，防止VEGF与血管内细胞表面受体VEGFR结合，减少新生血管生成和内皮细胞的增殖，防止肿瘤细胞转移。胡彬彬等^[11]^的研究表明，贝伐珠单抗能够改善肿瘤微血管结构，恢复血管通透性，降低组织间隙压力，保证药物顺利送到肿瘤组织，增加化疗药物达到肿瘤细胞的浓度，延缓培美曲塞方案的化疗耐药性，可强化化疗效果。

相关学者进一步发现，贝伐珠单抗联合培美曲塞治疗可能与调节机体的免疫应答机制相关。炎症微环境能通过多种细胞因子来促进肿瘤的发生、发展，参与肿瘤的转移等^[12-14]^。大量研究表明，IL-6^[15]^、IL-17^[16]^、IL-33^[17]^、TNF-α作为体内炎性介质，在肺癌的患者者，表达量明显升高，2015年鄢文等^[18]^研究发现贝伐珠单抗联合培美曲塞维持治疗后，较培美曲塞单药治疗的晚期非鳞非小细胞肺癌患者外周血IL-27表达有升高，可能与抑制了免疫逃逸相关。辛丽云等^[19]^、李英等^[20]^等发现贝伐珠单抗联合培美曲塞处理非小细胞肺癌移植瘤裸鼠后，IL-6、IL-17、IL-33、TNF-α等促瘤炎性相关因子表达水平下降，表明肿瘤预后得到改善。

近年研究表明，在非小细胞肺癌领域中，培美曲塞与贝伐珠单抗联用比单用两者任一药物都可延长PFS时间及OS时间。一项Ⅲ期临床AVAPERL研究^[21]^证实培美曲塞联合贝伐珠单抗较贝伐珠单抗单药维持治疗有更明显的无进展生存优势。国内多位学者^[14, 18, 19, 22]^也发现贝伐珠单抗联合培美曲塞加铂类化疗方案，在治疗非小细胞肺癌的近远期疗效上，要优于单独化疗对照组。本案例中患者的第一次及第二次应用培美曲塞时，未联用贝伐珠单抗，基于以上介绍的培美曲塞与贝伐珠单抗的协同作用，在第三次选择培美曲塞时，选择联用贝伐珠单抗进行综合治疗，且取得好的治疗效果，考虑与培美曲塞与贝伐珠单抗的协同作用有关。贝伐珠单抗是否可逆转培美曲塞的耐药性，目前尚无大宗的文献报道及证实，需要进一步研究。TS酶是胸苷酸合成酶，有关研究报道，TS低表达者，培美曲塞化疗效果好，且患者预后相对较好，而TS酶高表达者，对培美曲塞效果欠佳，且预后相对较差^[23, 24]^，该患者多次应用培美曲塞效果较好，可能存在TS酶低表达，遗憾的是因回顾性研究条件所限，该患者未行TS酶的检测。

患者的四线入组免疫治疗临床试验，IBI308（信达PD-1单抗）免疫治疗3个周期，评效PD（增大60%）。当时该患者应用在传统RECIST标准评效进展，未使用专门针对免疫治疗的iRECIST标准^[25]^，应研究组的要求，退出免疫治疗临床试验，所以当时不能判断是否为假性进展^[26]^，不能排除五线应用培美曲塞+卡铂+贝伐珠单抗时取得好的效果，是否会存在免疫治疗的后遗效应。目前在一些探索免疫治疗影响因素的研究中发现，肿瘤突变负荷大的肿瘤患者相对免疫治疗效果更佳，而化疗疗效相对较差。本案例患者免疫治疗的效果较差，但对化疗多次治疗有效，暗示我们如果关注患者的肿瘤突变负荷指标，可能更有探索价值。另外，免疫治疗3个周期虽然没给患者带来可见的近期疗效，但对减轻免疫逃逸对恢复这类患者的化疗耐药性，是否有积极意义，存在进一步的深入研究和探索空间。
